# Supplementary material for: Chromothripsis during telomere crisis is independent of NHEJ, and consistent with a replicative origin
Source: Genome Res. 2019 May;29(5):737–49. doi: 10.1101/gr.240705.118 (PMC6499312; doi:10.1101/gr.240705.118)
Supplement: Supplemental Material [file supp_gr.240705.118_Supplemental_file_1.zip › contigs/annotated_contigs/DB81/contig.2.DB81_length_507_mean_cov_12.1360946746.docx]

**DB81_length_507_mean_cov_12.1360946746**

```
TATTTATATGCAAGTGTTTCTGCTGGATACTAAGAATGATAAAAAAGAAATATGAGAAATGGTGTCTATATCAAGATATATTATAATCT
 >chr11:14777707-14777853 + E=3e-76
AGTTGGGGAGTGTATCAGTCAGGGTCCCAGCAAGAATAATACACTCCAATGGACTAG|AACGGATC|CAGACGGATCACGAGGTCAAGA
                                                                  >chr11:14872251-14872
GATCGAGACCATCCCGGCTAACACAGCGAAACCCCGTCTCTACTAAAAAAAAAAATAGCTGGGTGTGGTAGTCCCAGCTACTCCAAAGA
616 - E=4e-187
CTGAGGCAGGATAATGGCATGAACCTGGGGGGCGGAGCTTGCAGTAAGCCGAGAATGCCCCACTGCACTCCAGCCTGGGTGACAGAGCA

GGACTCCGTCTCAAAAAACAAAACAAAGCAAAACACAACACATATTTAGTTCCAAGTCCTAATGTTTAAAGCTATTGCAATTACAGTAT

CTATTTGTTGAGAACTCTTGCCTCTTTCATGCAAATTGTGACCTTAAAGGAATTTTATATTTTA
```
